# Supplementary material for: Loss of Kmt2c or Kmt2d primes urothelium for tumorigenesis and redistributes KMT2A–menin to bivalent promoters
Source: Nat Genet. 2025 Jan 13;57(1):165–79. doi: 10.1038/s41588-024-02015-y (PMC11735410; doi:10.1038/s41588-024-02015-y)
Supplement: Supplementary file 1 — Supplementary Figs. 1–10. [file 41588_2024_2015_MOESM1_ESM.pdf]

nature genetics

Supplementary information https://doi.org/10.1038/s41588-024-02015-y

Loss of *Kmt2c* or *Kmt2d* primes urothelium for tumorigenesis and redistributes KMT2A–menin to bivalent promoters

In the format provided by the authors and unedited

Supplementary Fig. 1 Characterization of urothelial cell clusters in scRNA-seq.

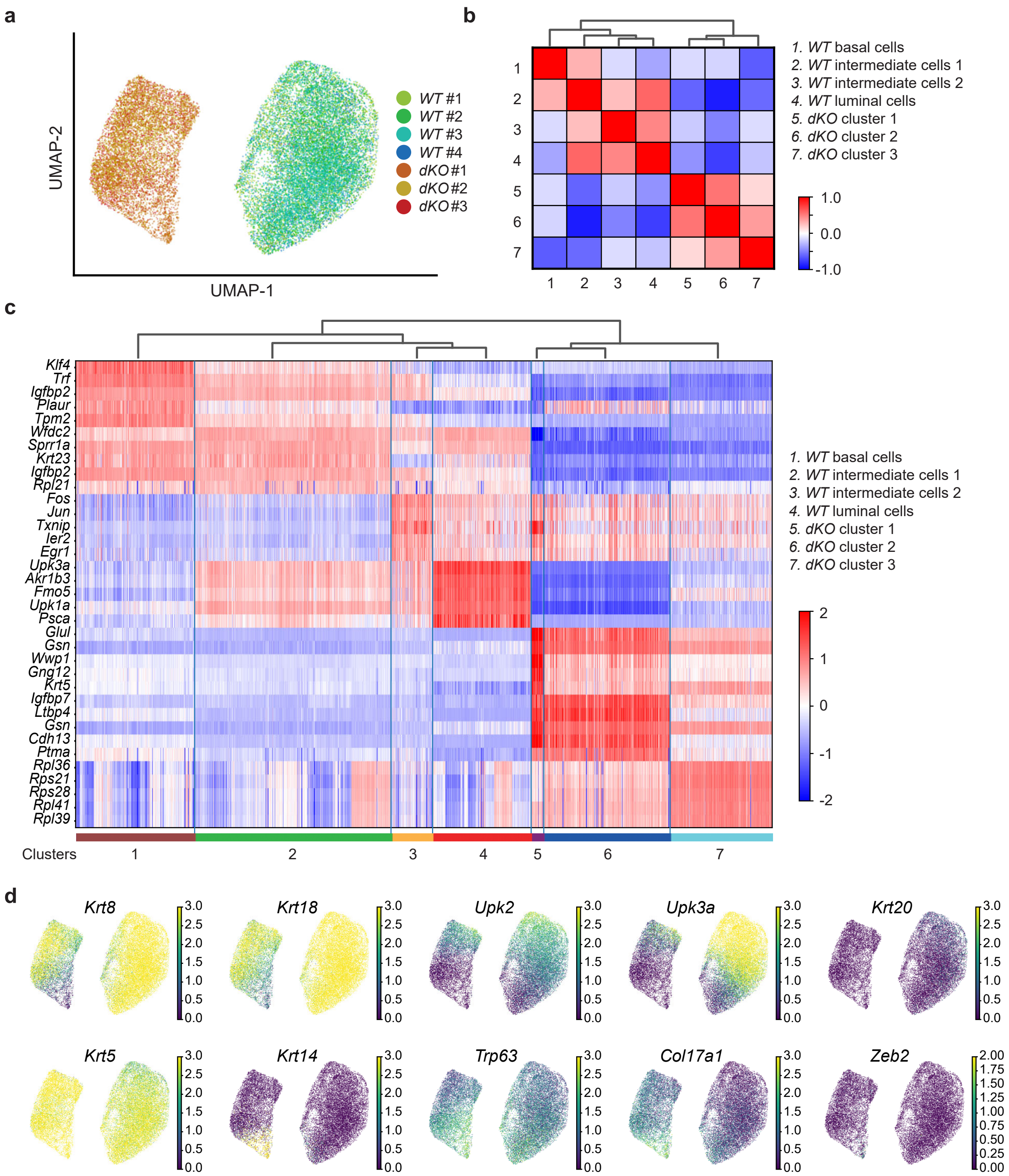

Supplementary Fig. 1: Characterization of urothelial cell clusters in scRNA-seq.

a, UMAP plots color-coded by mouse showing the consistency of biological replicates in *WT* (n=4 mice) and *dKO* (n=3 mice) groups.

b, Pearson correlation coefficient of clusters identified in *WT* and *dKO* groups.

c, Heatmap depicting expression of cluster markers identified by differential expression analysis (cluster vs. the rest). Color bar: z-score of Log_2(CPM+1). Red color corresponds to positive correlation, while blue color corresponds to negative correlation.

d, UMAP color-coded by expression of representative basal, luminal, and EMT markers.

Supplementary Fig. 2 Heatmap of differentially expressed genes from scRNA-seq.

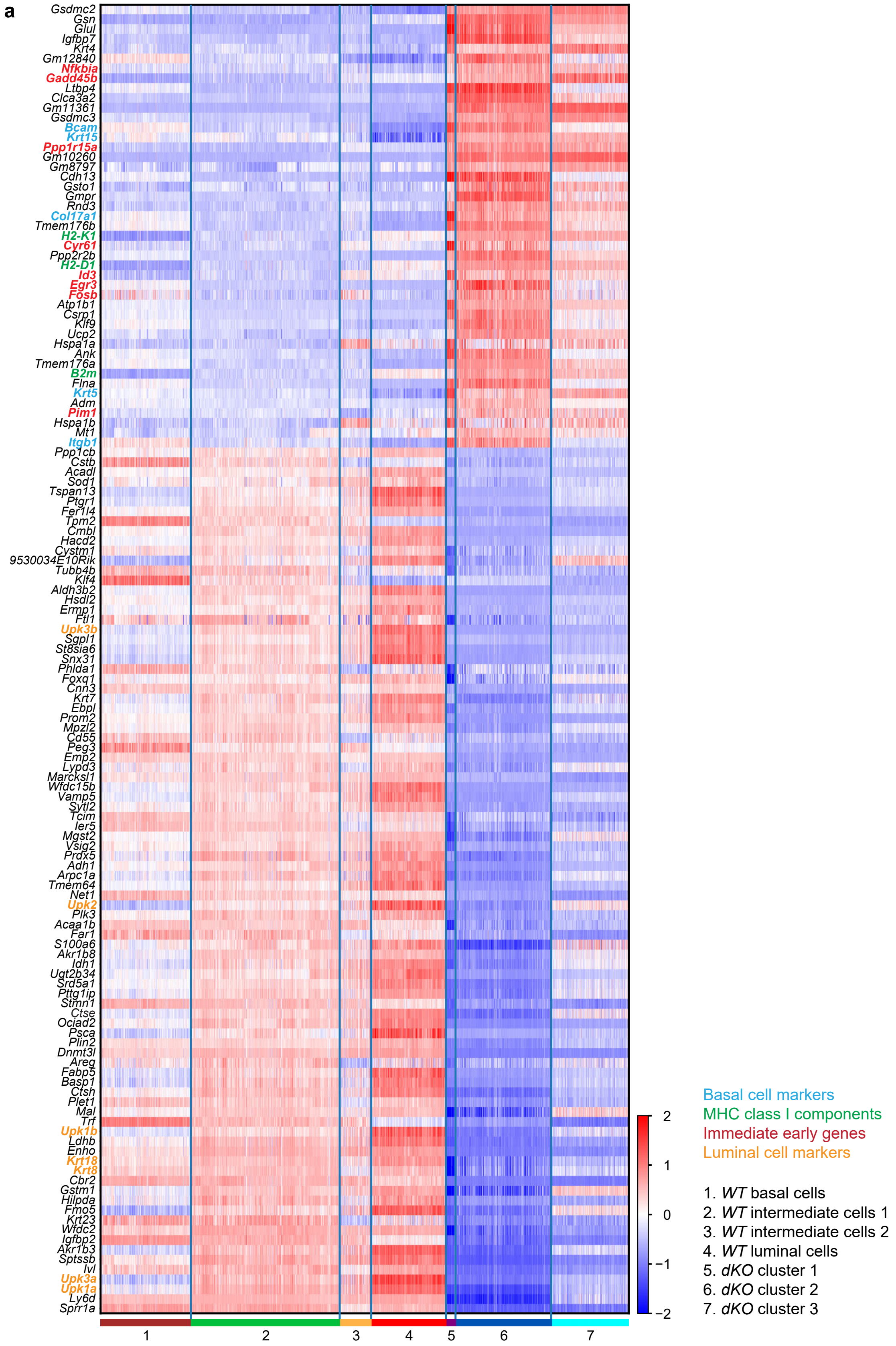

Supplementary Fig. 2: Heatmap of differentially expressed genes from scRNA-seq.

a, Single transcriptomes from each condition were pooled together. Differentially expressed genes were identified with ANOVA. Compared to *WT* group, 45 genes were significantly upregulated in *dKO* group, while 88 genes were significantly downregulated in *dKO* group (absolute fold change >1.5, FDR<0.05). *Kmt2c/d dKO* cells expressed higher basal cell markers (e.g., *Bcam*, *Krt15*, *Krt5*, *Col17a1*, *Itgb1*), MHC class I components (e.g., *B2m*, *H2-D1*, *H2-K1*), and immediate early genes (IEGs) (e.g., *Egr3*, *Cyr61*), while they expressed lower luminal cell markers (e.g., Uroplakins, *Krt8*, *Krt18*).Color bar: z-score of Log_2(CPM+1).

Supplementary Fig. 3 Transcriptional alterations after *Kmt2c/d* knockout.

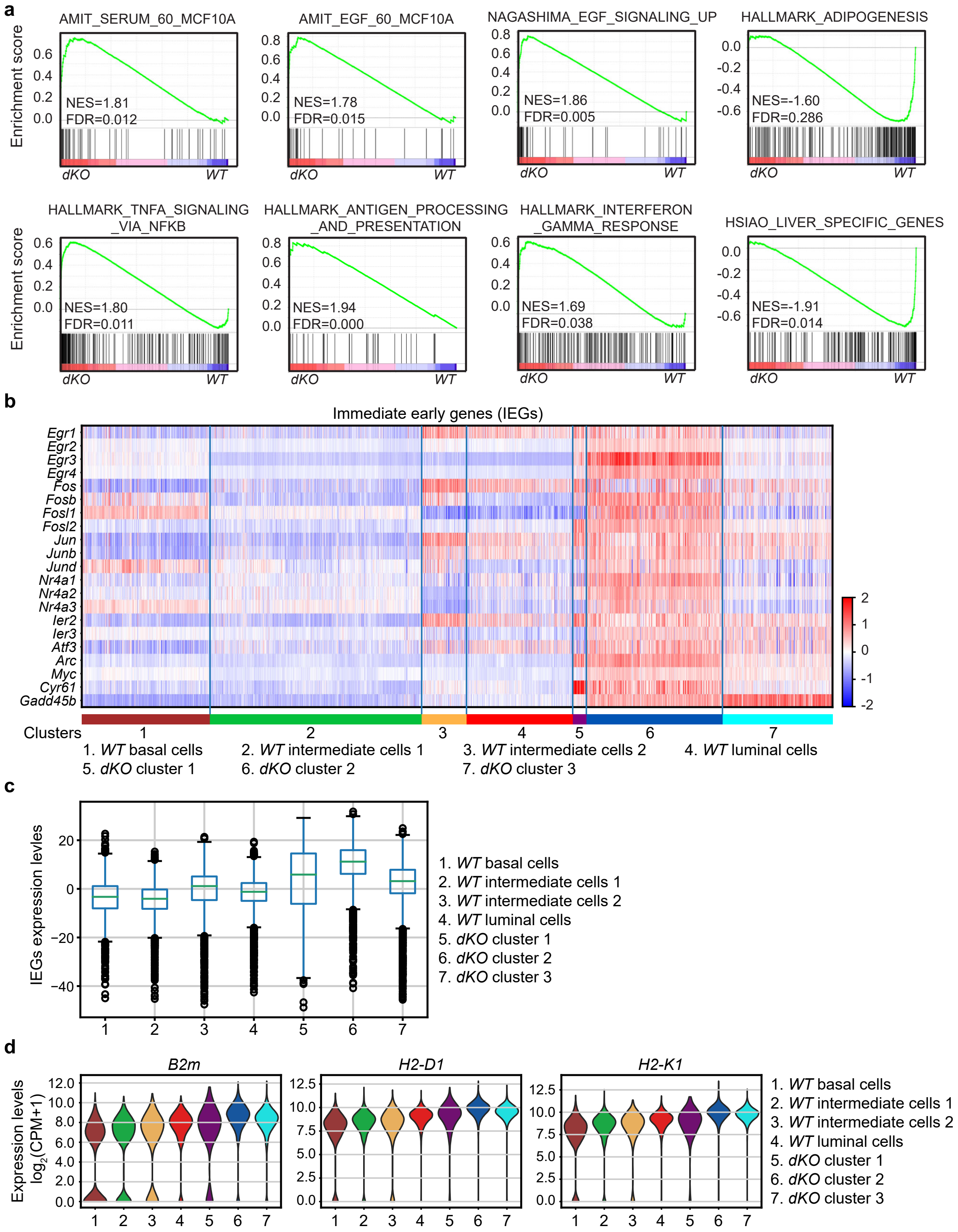

Supplementary Fig. 3: Transcriptional alterations after *Kmt2c/d* knockout.

a, GSEA analysis of single-cell transcriptomes indicating the positive enrichment of gene sets associated with EGF/serum stimulation and inflammation, and negative enrichment of gene sets associated with differentiation.

b, Heatmap of imputed expression of 21 well-known IEGs in cell clusters from scRNA-seq. Color bar: z-score of log_2(CPM+1).

c, Sum of z-score of IEGs in each single-cell transcriptome from (b). The center line represents the median; the box limits represent the upper and lower quartiles; the whiskers represent 1.5x the interquartile range.

d, Violin plots showing normalized expression levels of *B2m*, *H2-D1* and *H2-K1* in *WT* and *dKO* clusters.

Supplementary Fig. 4 *Kmt2c/d* deletion decreases enhancer activities and KMT2A/SET1A deposition.

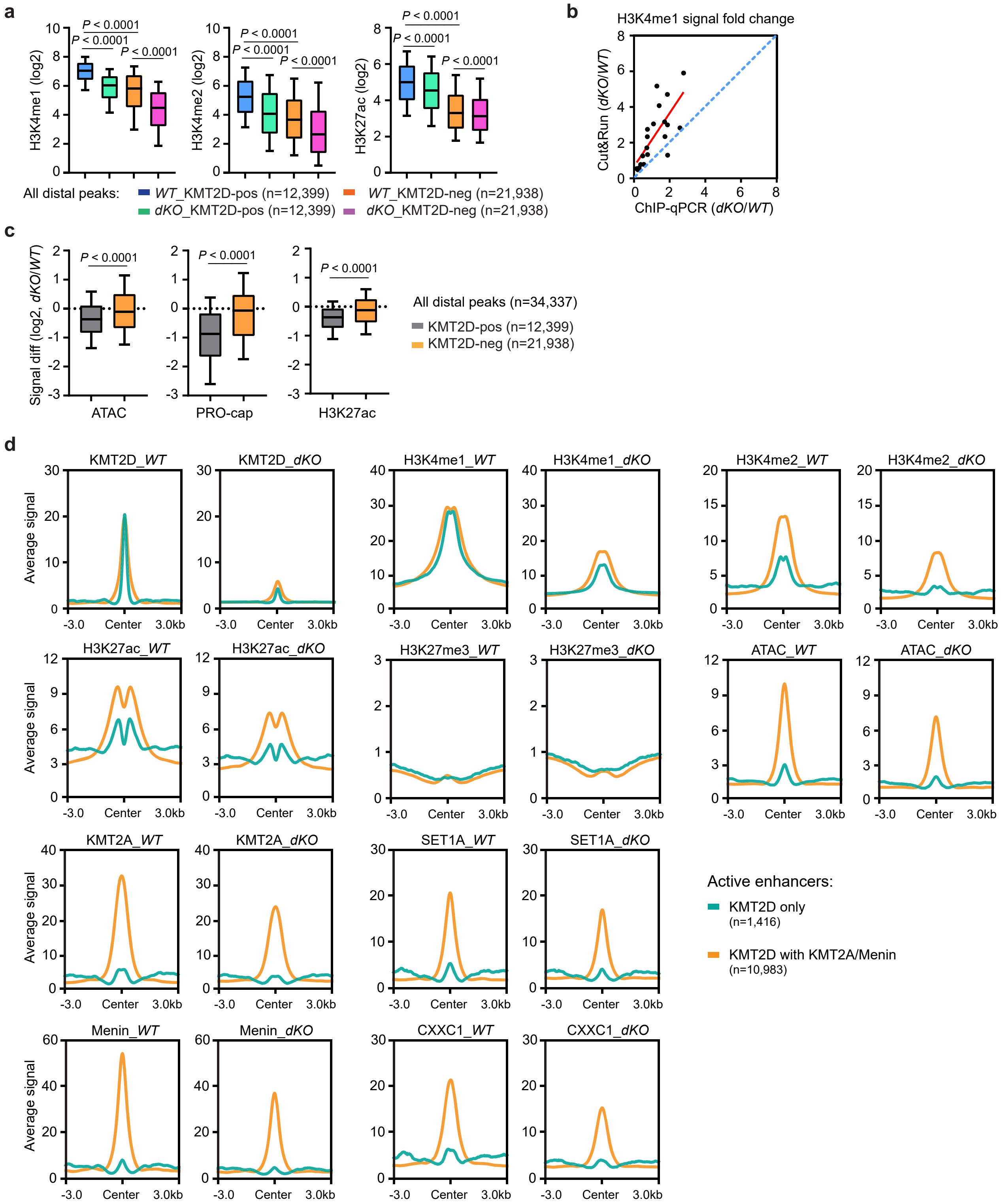

Supplementary Fig. 4: *Kmt2c/d* deletion decreases enhancer activities and KMT2A/SET1A deposition.

a, H3K4me1, H3K4me2, and H3K27ac signal at KMT2D-positive and KMT2D-negative enhancers in *WT* and *dKO* urothelial cells. The center line represents the median; the box limits represent the upper and lower quartiles; the minimum and maximum whiskers represent 10 and 90 percentile. Data were analyzed with two-tailed t-test.

b, Correlation of H3K4me1 enrichment in Cut&Run and ChIP-qPCR at 20 different loci including both enhancers and promoters. Signal in Cut&Run (*dKO*/*WT*) exhibited an average of 2.28-fold more changes compared to ChIP-qPCR. The Cut&Run of H3K4me1 in *dKO* cells was further normalized with this value.

c, Log2 fold change of ATAC-seq, PRO-cap, and H3K27ac at KMT2D-positive and KMT2D-negative enhancers in *WT* and *dKO* urothelial cells. The center line represents the median; the box limits represent the upper and lower quartiles; the minimum and maximum whiskers represent 10 and 90 percentile. Data were analyzed with two-tailed t-test.

d, Aggregation plots comparing the average enrichment of KMT2D, H3K4me1, H3K4me2, H3K27ac, H3K27me3, ATAC-seq, KMT2A, Menin, SET1A, and CXXC1 at 2 subgroups of KMT2D positive enhancers in *WT* and *dKO* urothelial cells.

Supplementary Fig. 5 Heatmap of individual replicate at KMT2D-bound active enhancers.

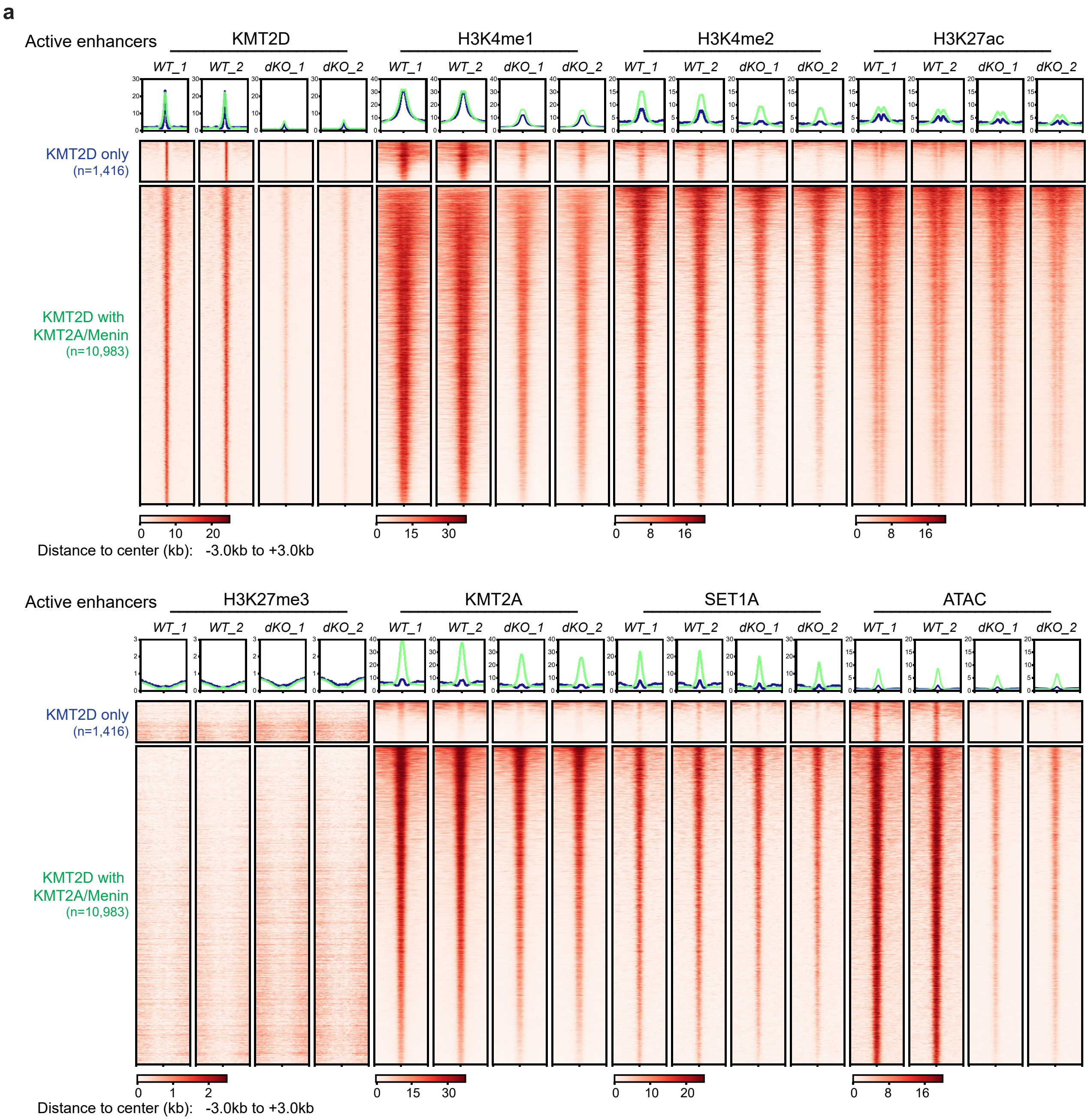

Supplementary Fig. 5: Heatmap of individual replicate at Kmt2d-bound active enhancers.

a, Heatmap showing enrichment of individual replicate of KMT2D, H3K4me1, H3K4me2, H3K27ac, H3K27me3, KMT2A, SET1A, and ATAC-seq in *WT* and *dKO* urothelial cells.

Supplementary Fig. 6 Representative IGV tracks showing decreased enhancer activities after *Kmt2c/d* deletion.

a

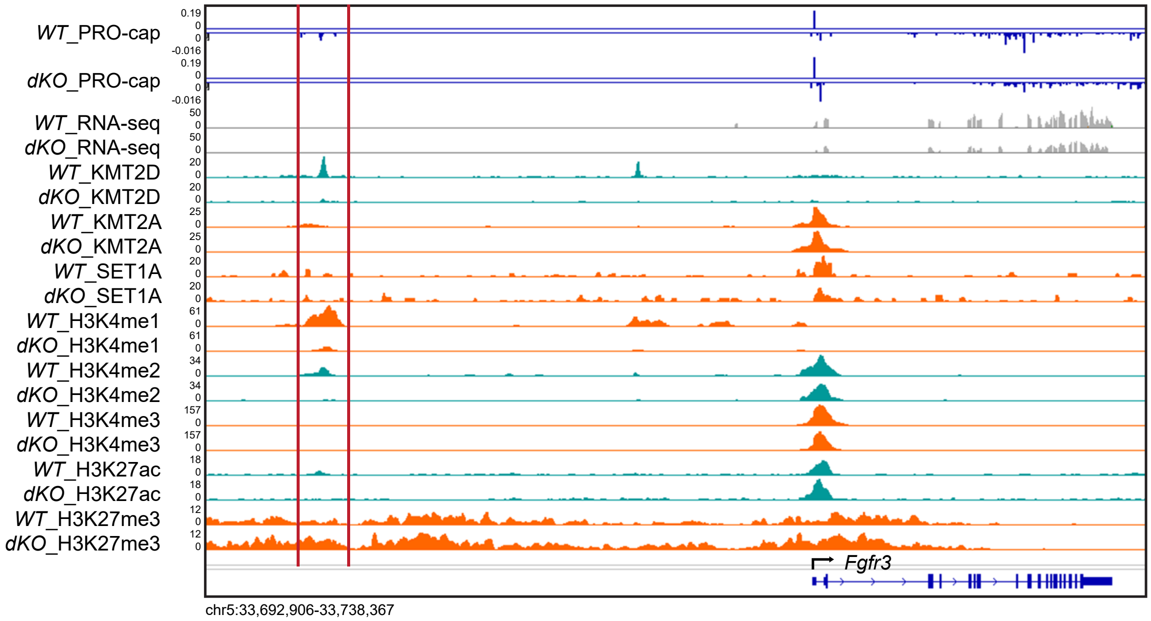

b

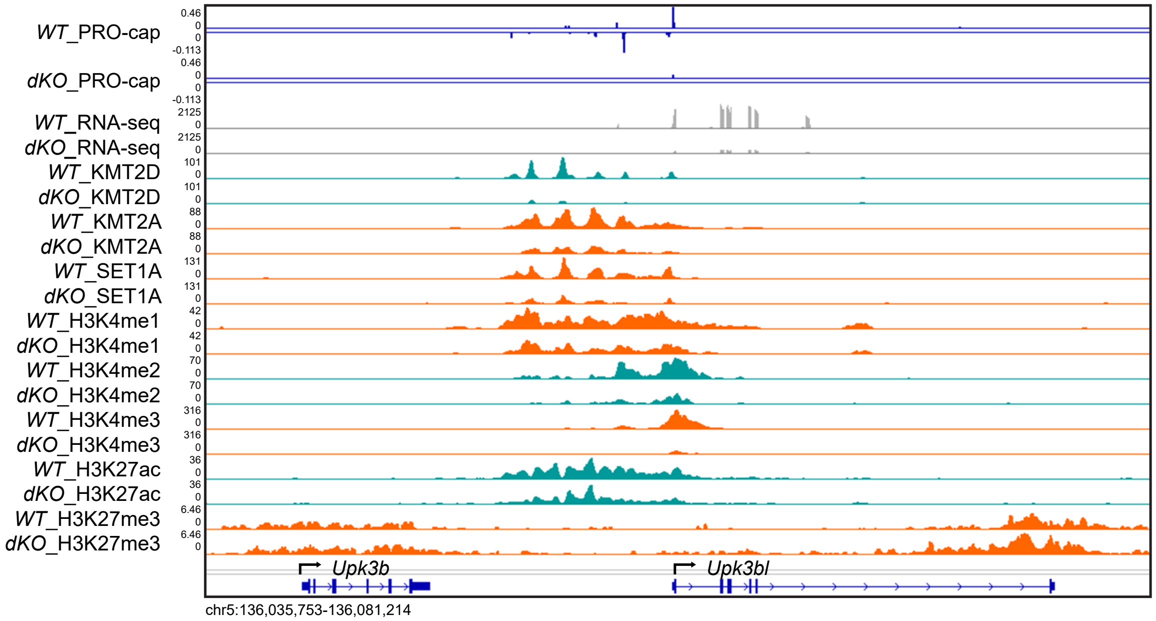

c

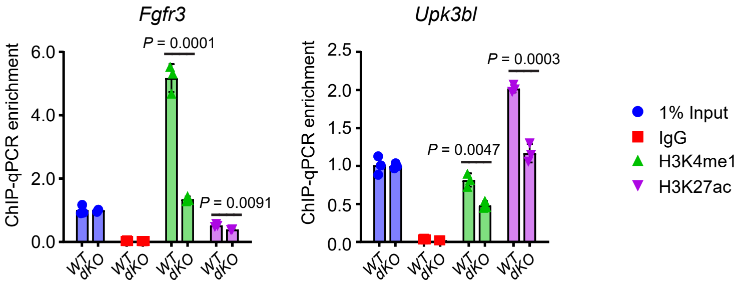

Supplementary Fig. 6: Representative IGV tracks showing decreased enhancer activities after *Kmt2c/d* deletion.

a and **b**, Representative IGV tracks of PRO-cap, RNA-seq, KMT2D, KMT2A, SET1A, H3K4me1, H3K4me2, H3K4me3, H3K27ac, and H3K27me3 depositions on *Fgfr3* and *Upk3bl* in *WT* and *dKO* urothelial cells. The enhancers are marked by PRO-cap bidirectional transcripts, presence of H3K4me1 and H3K27ac that declined after *Kmt2c/d* deletion. Tracks within the two red lines showed one of the KMT2D-bound active enhancers.

c, ChIP-qPCR of H3K4me1 and H3K27ac on KMT2D positive enhancer peaks shown in a and b (representative of n=2 independent experiments). Data were presented as mean±SD and analyzed with two-tailed t-test.

Supplementary Fig. 7 *Kmt2c/d* deiciency results in activation of CpG-high promoters.

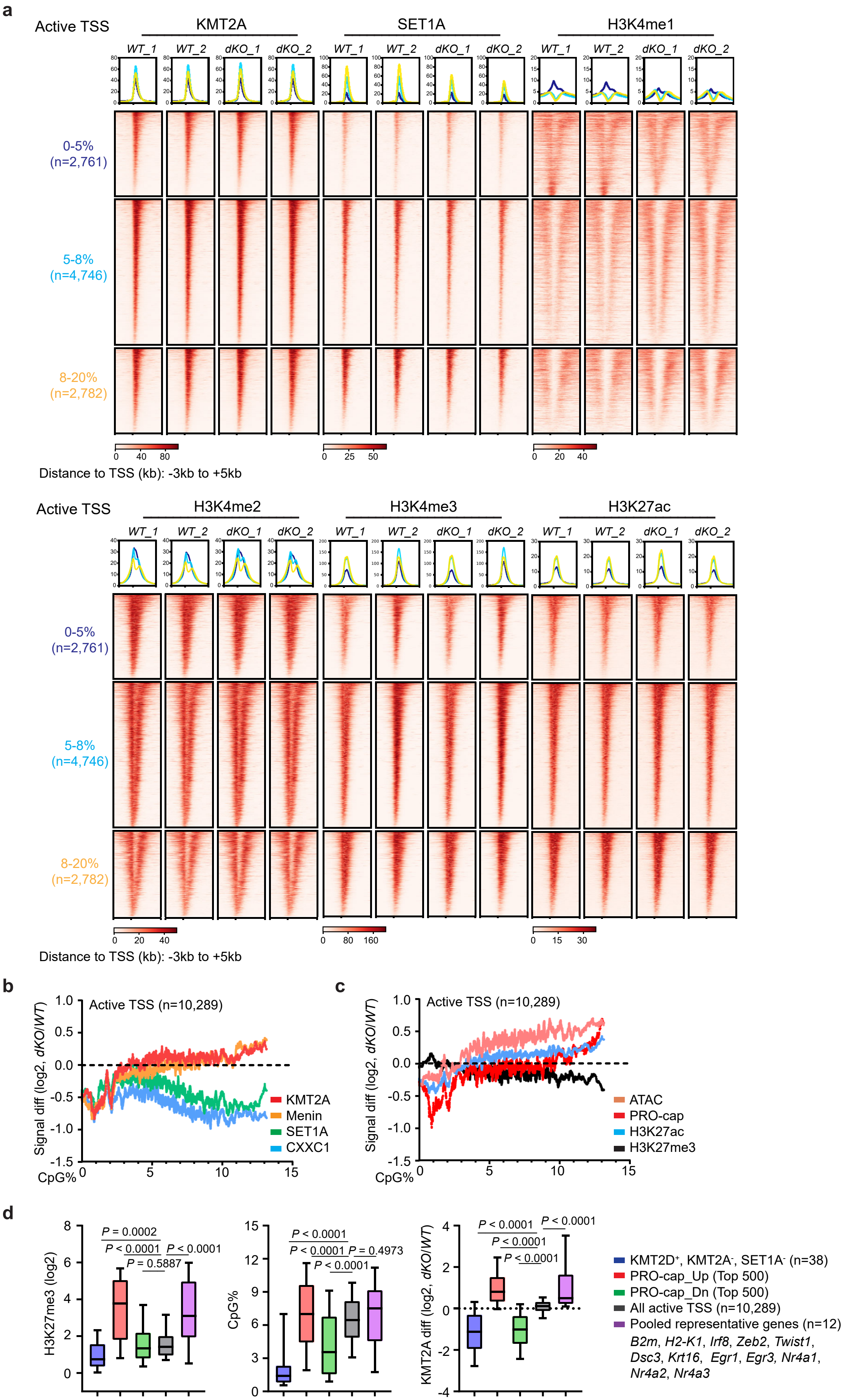

Supplementary Fig. 7: *Kmt2c/d* deficiency results in activation of CpG-high promoters.

a, Heatmap showing enrichment of individual replicate of KMT2A, SET1A, H3K4me1, H3K4me2, H3K4me3, and H3K27ac in *WT* and *dKO* urothelial cells.

b and **c**, Log2 fold change of KMT2A, Menin, SET1A, CXXC1, ATAC-seq, H3K27ac, H3K27me3, and PRO-cap signal with respect to CpG content. Active TSS was ranked by CpG% within proximal KMT2D, KMT2A/Menin, or SET1A/CXXC1 peaks. Bin size of 100 was then used to smoothen the log2 fold change of signal.

d, H3K27me3, CpG%, and KMT2A log2 fold change at KMT2D-only TSS, top 500 PRO-cap up- or down- regulated TSS, all TSS, and pooled TSS of representative genes (n=12). The center line represents the median; the box limits represent the upper and lower quartiles; the minimum and maximum whiskers represent 10 and 90 percentile. Data were analyzed with two-tailed t-test.

Supplementary Fig. 8 Representative IGV tracks showing increased KMT2A deposition at promoter regions.

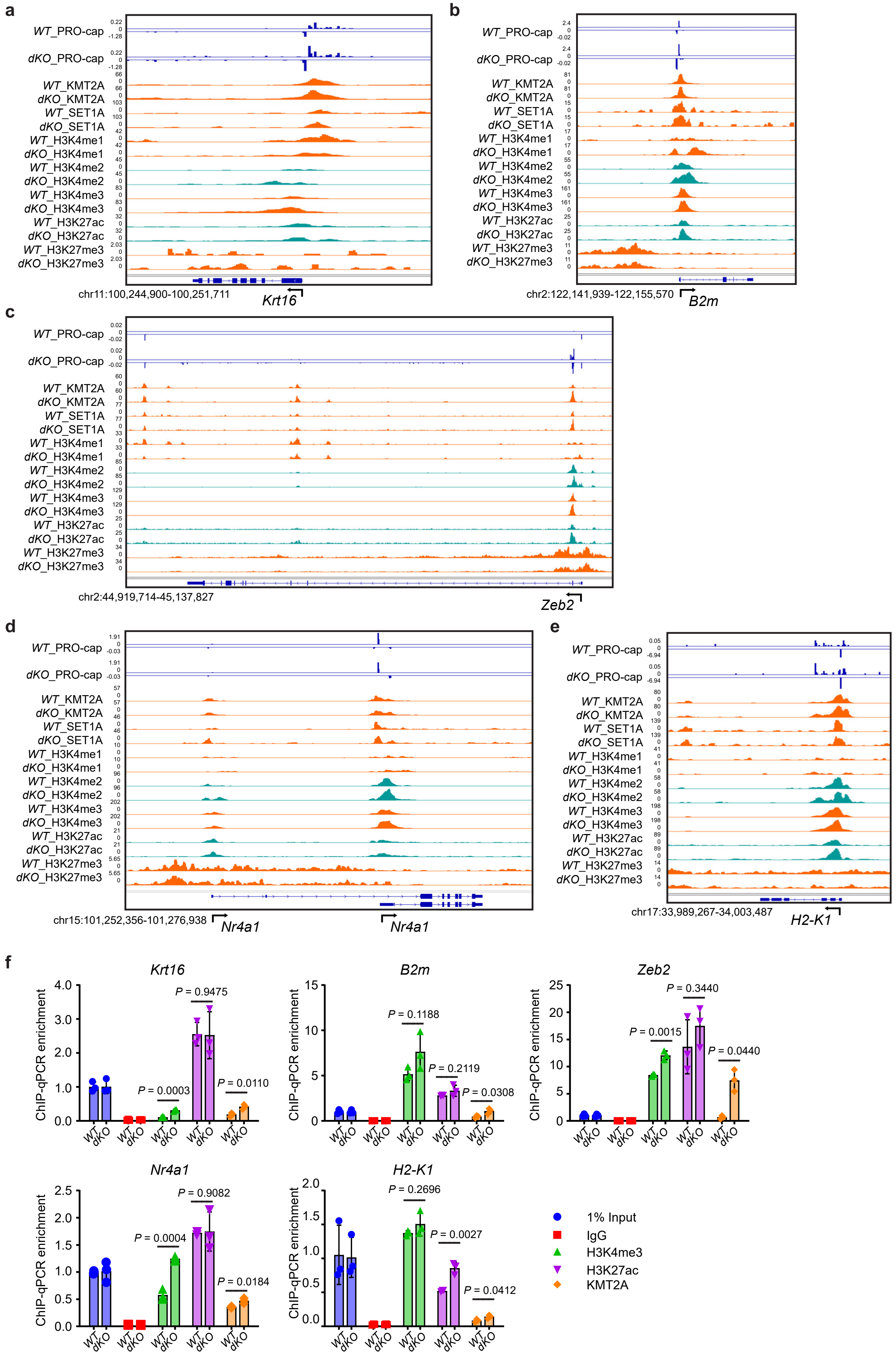

Supplementary Fig. 8: Representative IGV tracks showing increased KMT2A deposition at promoter regions.

a-e, Representative IGV tracks of PRO-cap, KMT2A, SET1A, H3K4me1, H3K4me2, H3K4me3, H3K27ac, and H3K27me3 on TSS of basal markers (e.g., *Krt16*), EMT markers (e.g., *Zeb2*), IEGs (e.g., *Nr4a1*), and MHC class I molecules (e.g., *B2m*, *H2-K1*).

f, ChIP-qPCR of H3K4me3, H3K27ac and Kmt2a at promoter regions shown in a-e (representative of n=2 independent experiments). Data were presented as mean±SD and analyzed with two-tailed t-test.

Supplementary Fig. 9 Validation of *Kmt2c*, *Kmt2d*, and *Pten* knockout in mouse bladder and ureter tissues.

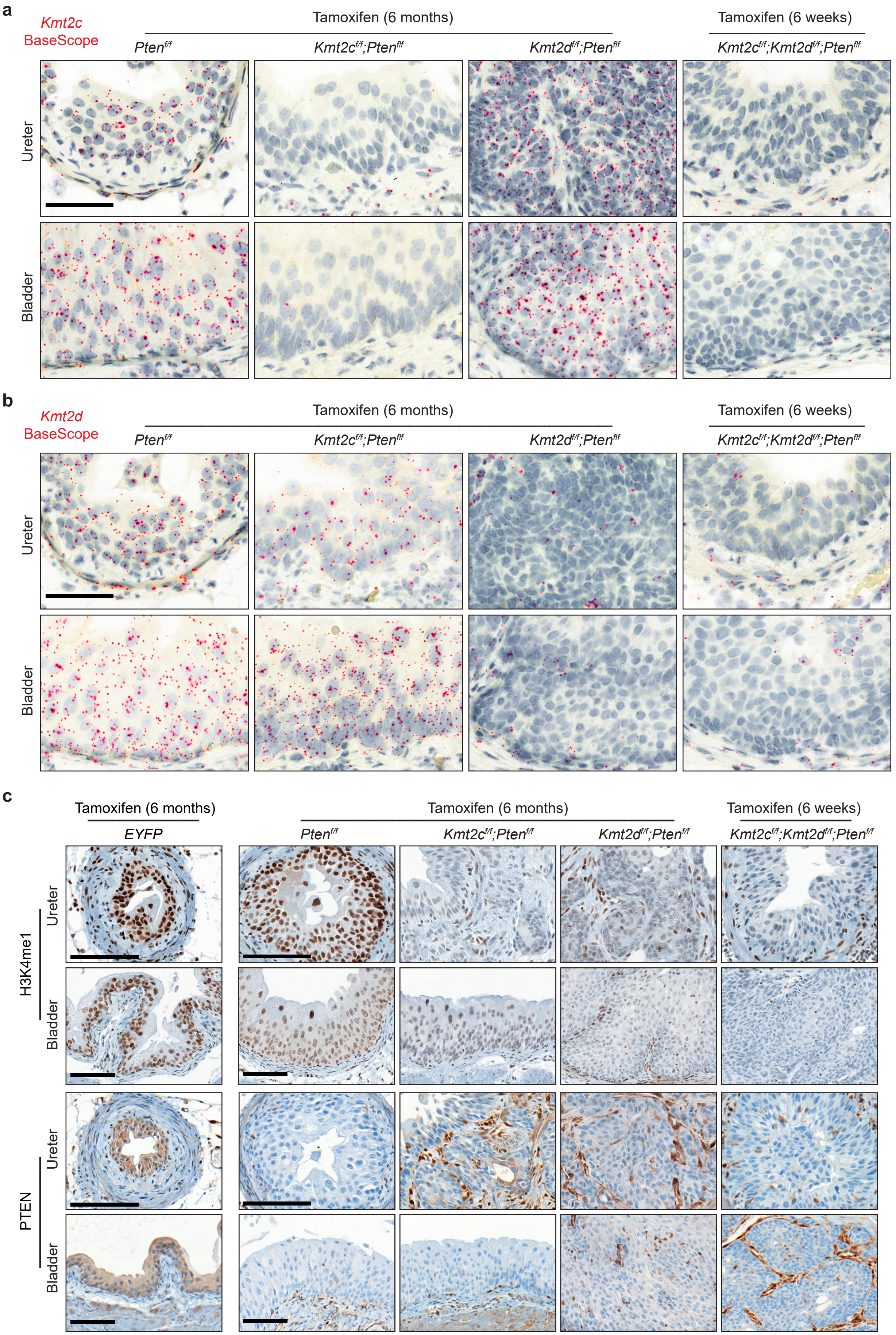

Supplementary Fig. 9: Validation of *Kmt2c*, *Kmt2d*, and *Pten* knockout in mouse bladder and ureter tissues.

a and **b**, Representative BaseScope staining with probes targeting *Kmt2c* or *Kmt2d* floxed exons (n=3 mice in each group). Scale bar, 50µm.

c, Representative histological staining of H3K4me1 and PTEN validating the successful deletions of *Kmt2c/d* and *Pten* in urothelial cells (n=3 mice in each group). Scale bar, 100µm.

Supplementary Fig. 10 Histological characterization of BBN-induced urothelial carcinoma models.

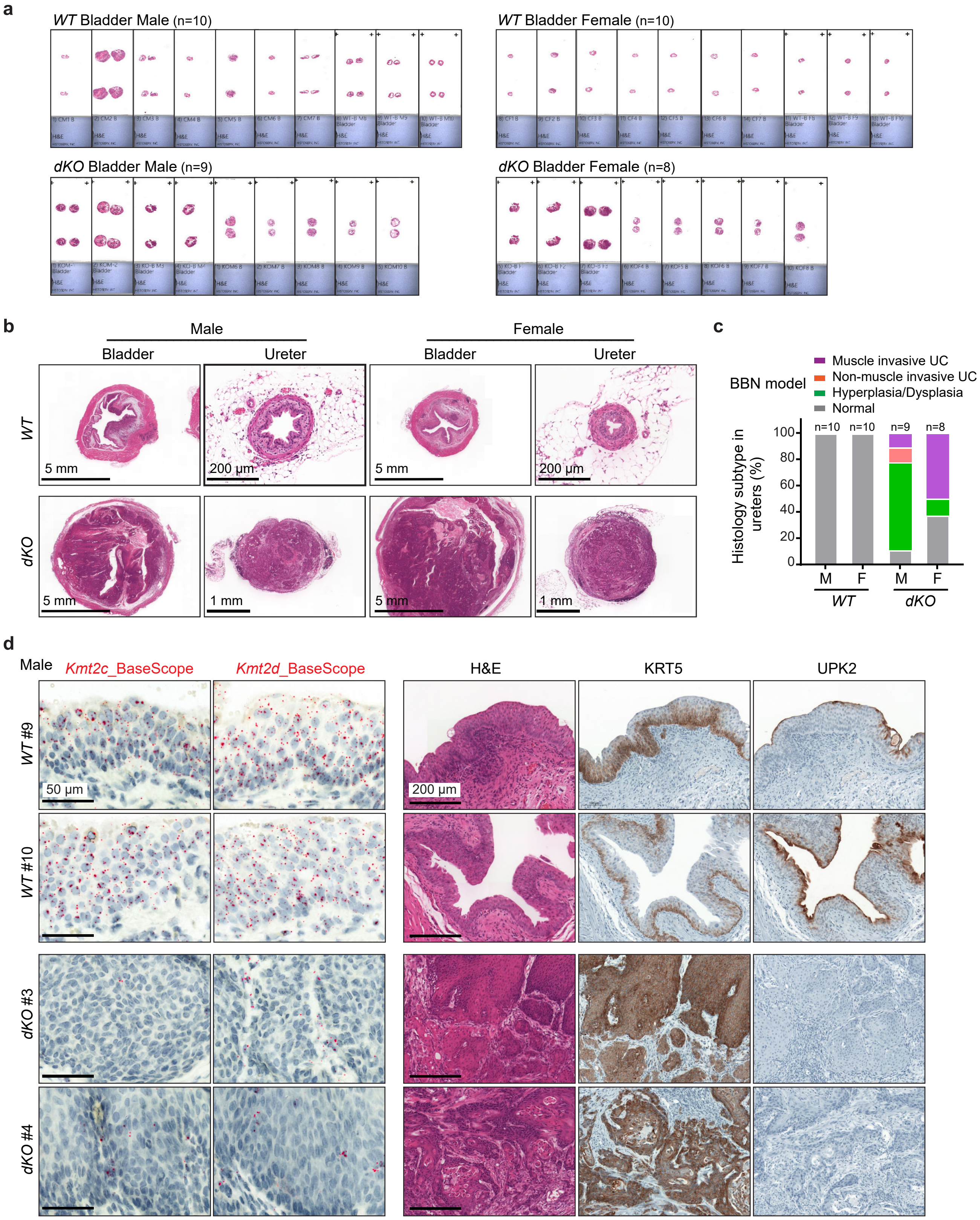

Supplementary Fig. 10: Histological characterization of BBN-induced urothelial carcinoma models.

a, H&E staining of all bladder tissue sections from BBN-induced urothelial carcinoma models. Note that one case of bladder tumorigenesis in the male *dKO* group was confirmed by necropsy, but tissues were not collected for histological analysis.

b, Representative H&E staining of bladder and ureter sections in *WT* (n=20 mice) and *dKO* (n=17 mice) groups. Scale bars were indicated in the figures.

c, Histological subtypes of ureter urothelium in male and female BBN models.

d, Representative H&E, *Kmt2c/d* BaseScope (n=2 mice), KRT5 IHC (n=6 mice), and UPK2 IHC (n=6 mice) staining in bladder tissue sections collected from male *WT* and *dKO* mice. Scale bar is 50 μm for BaseScope and 200μm for HE and IHC staining.
