# Supplementary material for: Loss of Kmt2c or Kmt2d primes urothelium for tumorigenesis and redistributes KMT2A–menin to bivalent promoters
Source: Nat Genet. 2025 Jan 13;57(1):165–79. doi: 10.1038/s41588-024-02015-y (PMC11735410; doi:10.1038/s41588-024-02015-y)
Supplement: Supplementary file 2 — Reporting Summary [file 41588_2024_2015_MOESM2_ESM.pdf]

nature portfolio

Corresponding author(s): Yu Chen

Last updated by author(s): 10/2/2024

Reporting Summary

Nature Portfolio wishes to improve the reproducibility of the work that we publish. This form provides structure for consistency and transparency in reporting. For further information on Nature Portfolio policies, see our Editorial Policies and the Editorial Policy Checklist.

Statistics

For all statistical analyses, confirm that the following items are present in the figure legend, table legend, main text, or Methods section.

n/a	Confirmed	
☐	☒	The exact sample size (*n*) for each experimental group/condition, given as a discrete number and unit of measurement
☐	☒	A statement on whether measurements were taken from distinct samples or whether the same sample was measured repeatedly
☐	☒	The statistical test(s) used AND whether they are one- or two-sided<br>*Only common tests should be described solely by name; describe more complex techniques in the Methods section.*
☒	☐	A description of all covariates tested
☐	☒	A description of any assumptions or corrections, such as tests of normality and adjustment for multiple comparisons
☐	☒	A full description of the statistical parameters including central tendency (e.g. means) or other basic estimates (e.g. regression coefficient) AND variation (e.g. standard deviation) or associated estimates of uncertainty (e.g. confidence intervals)
☐	☒	For null hypothesis testing, the test statistic (e.g. *F*, *t*, *r*) with confidence intervals, effect sizes, degrees of freedom and *P* value noted<br>*Give P values as exact values whenever suitable.*
☒	☐	For Bayesian analysis, information on the choice of priors and Markov chain Monte Carlo settings
☒	☐	For hierarchical and complex designs, identification of the appropriate level for tests and full reporting of outcomes
☐	☒	Estimates of effect sizes (e.g. Cohen's *d*, Pearson's *r*), indicating how they were calculated

Our web collection on statistics for biologists contains articles on many of the points above.

Software and code

Policy information about availability of computer code

Data collection	Histology and organoids: Mirax Digital Slide Scanner, Leica TCS SP5 upright confocal microscope, Nikon ECLIPSE Ti2 inverted microscope;<br>Flow cytometry: BD LSRFortessa, BD FACSymphony S6 Cell Sorter;<br>Luminesent imaging: IVIS Spectrum imaging system;<br>scRNA-seq: Illumina NovaSeq platform S4 flow cell;<br>RNA-seq, PRO-cap, ChIP-seq, Cut&Run, and ATAC-seq: Illumina platforms HiSeq4000 and NovaSeq6000, ;<br>PCR reaction: Applied Biosystems QuantStudio 7 Flex Real-Time PCR machine;<br>Western blot: Amersham ImageQuant 800 biomolecular imager;<br>Histone PTMs: Thermo Scientific TSQ Quantum Ultra mass spectrometer, UltiMate 3000 Dionex nano-liquid chromatography;
Data analysis	Flow cytometry data were analyzed with BD FACSDiva software v6.2 and FlowJo 10.7.1;<br>IHC and BaseScope quantification: QuPath-0.4.4;<br>scRNA-seq data were processed with Cell Ranger (7.0.0). True cells were identified using scCB2 package (1.14.0). Putative doublets were detected and filtered out using doublet detection package (4.2). Downstream analyses and figure plotting were performed using Scanpy (1.6.1). Differentially expressed genes were compared with MAST package (1.30.0). Imputated data were used to generate heatmap using MAGIC (3.0.0) package.<br>RNA-seq data were processed with STAR (2.7.10b);<br>PRO-cap data were mapped with STAR (2.7.10b). Peaks were called with PINTS 1.1.8 (pints_caller). Bigwig files of plus and minus strand alignments were generated with PINTS 1.1.8 (pints_visualizer);<br>GSEA analyses were performed using JAVA GSEA 4.1.0 program;<br>TCGA human MIBC data were analyzed with ssGSEA v4.0;<br>Data from ATAC-seq, ChIP-seq, Cut&Run were trimmed with trim_galore and mapped to GRCm38(mm10) using bowtie2 (2.4.5). Duplicates

were marked with samblaster (0.1.26). Mapping quality was analyzed with qualimap (2.2.2-dev). Peaks were called using MACS3 (3.0.0). Bigwig files were generated with bamCoverage (3.5.1). Read counts were measured with featureCounts (v2.0.1); Heatmap and aggregation plots were generated using deepTools (3.5.1).
In KMT2D ChIP-seq, Sicer2 (1.0.3) (sicer_df) was used to call differential peaks between WT and dKO conditions;
Homer (4.11.1) was employed to merge (Homer mergePeaks) and annotate (Homer annotatepeaks.pl) peaks from PRO-cap, ATAC-seq, ChIP-seq, and Cut&Run;
ChromHMM (v1.25) LearnModel was performed to investigate chromatin state in WT and dKO cells. ChromHMM OverlapEnrichment was conducted to compare enrichment of chromatin states at given coordinates.
Histone PTMs were analyzed with Skyline.

For manuscripts utilizing custom algorithms or software that are central to the research but not yet described in published literature, software must be made available to editors and reviewers. We strongly encourage code deposition in a community repository (e.g. GitHub). See the Nature Portfolio guidelines for submitting code & software for further information.

Data

Policy information about availability of data

All manuscripts must include a data availability statement. This statement should provide the following information, where applicable:

- Accession codes, unique identifiers, or web links for publicly available datasets
- A description of any restrictions on data availability
- For clinical datasets or third party data, please ensure that the statement adheres to our policy

Raw sequencing data are publicly available from Gene Expression Omnibus: GSE180947, GSE236370, and GSE264514. Raw mass spectrometry data of histone post translational modifications are available via ProteomeXchange with identifier PXD056439. All other data supporting the findings of this study are available upon request from the corresponding authors.
This study did not use custom code or software. All code used in the study has been published and has been cited in the relevant section of the Methods.

Research involving human participants, their data, or biological material

Policy information about studies with human participants or human data. See also policy information about sex, gender (identity/presentation), and sexual orientation and race, ethnicity and racism.

Reporting on sex and gender	N/A
Reporting on race, ethnicity, or other socially relevant groupings	N/A
Population characteristics	N/A
Recruitment	N/A
Ethics oversight	N/A

Note that full information on the approval of the study protocol must also be provided in the manuscript.

Field-specific reporting

Please select the one below that is the best fit for your research. If you are not sure, read the appropriate sections before making your selection.

☒ Life sciences ☐ Behavioural & social sciences ☐ Ecological, evolutionary & environmental sciences

For a reference copy of the document with all sections, see nature.com/documents/nr-reporting-summary-flat.pdf

Life sciences study design

All studies must disclose on these points even when the disclosure is negative.

Sample size	Sample sizes were not predetermined by any statistical methods. In our experiments, sample sizes were similar to prior studies (PMID: 37084735) and indicated in the figures or figure legends.
Data exclusions	No data were excluded from the analyses.
Replication	Experiments were successfully repeated with a minimum of two independent experiments. In most cases, we performed multiple experiments to address the same scientific question.
Randomization	For the genetically engineered mouse models, we randomly gave tamoxifen to both male and female mice with the matched ages.<br>In the therapeutic experiments, tumor-bearing mice were randomized before the treatment to ensure comparable tumor sizes among groups at the beginning timepoint.

Blinding	Next-generation sequencing data were obtained and analyzed unbiasedly in this study. Mouse treatment and tumor measurements were performed by the same person blinded to the therapeutic effects. Statistics of Ki67 positivity were conducted with the whole bladder sections using QuPath program.

Reporting for specific materials, systems and methods

We require information from authors about some types of materials, experimental systems and methods used in many studies. Here, indicate whether each material, system or method listed is relevant to your study. If you are not sure if a list item applies to your research, read the appropriate section before selecting a response.

Materials & experimental systems

n/a	Involved in the study
☐	☒ Antibodies
☒	☐ Eukaryotic cell lines
☒	☐ Palaeontology and archaeology
☐	☒ Animals and other organisms
☒	☐ Clinical data
☒	☐ Dual use research of concern
☒	☐ Plants

Methods

n/a	Involved in the study
☐	☒ ChIP-seq
☐	☒ Flow cytometry
☒	☐ MRI-based neuroimaging

Antibodies

Antibodies used

KRT5, #905501, Biolegend (1:500 in IHC, 1:400 in IF, 1:400 in Flow)
KRT5, #905901, Biolegend (1:400 in IF)
KRT7, #ab181598, lot# GR3214132-2, Abcam (1:1000 in IHC)
KRT8, #904801, Biolegend (1:400 in IF, 1:400 in Flow)
KRT14, #906004, Biolegend (1:400 in IF)
KRT20, #M7091, clone# KS20.8, lot# 20046893, Dako Omnis (1:500 in IF)
UPK2, #ab213655, lot# GR284813-6, Abcam (1:500 in IHC and IF)
PTEN, #9188, clone# D4.3, lot# 6, Cell Signaling Technology (1:100 in IHC, 1:2000 in WB)
GFP, #2956, clone# D5.1, lot# 6, Cell Signaling Technology (1:200 in IHC)
Ki-67, #ab16667, Abcam (1:100 in IHC, 1:100 in IF)
SMA, #ab5694, lot# GR3356867-5, Abcam (1:1000 in IHC)
p-EGFR Y845, #2231, lot# 8, Cell Signaling Technology (1:50 in IHC)
H-2Kb/Db-APC, #114614, clone# 28-8-6, lot# B312433, Biolegend (1:200 in flow cytometry)
PD-L1-APC, #124311, clone# 10F.9G2, lot# B357778, Biolegend (1:200 in flow cytometry)
EpCAM-APC, #17579180, clone# G8.8, lot# 2202308, Thermo Fisher Scientific (1:200 in flow cytometry)
H3K4me1, #710795, lot# 1998633, Thermo Fisher Scientific (1ug per 250k cells in Cut&Run)
H3K4me1, #5326, clone# D1A9, lot# 5, Cell Signaling Technology (1:100 in IHC)
H3K4me2, #710796, lot# 2059496, Thermo Fisher Scientific (1ug per 250k cells in Cut&Run)
H3K4me3, #PA57-27029, lot# TI4042096A, Thermo Fisher Scientific (1ug per 250k cells in Cut&Run)
H3K27ac, #ab4729, lot# GR3231887-1, Abcam (2ug per 10million cells in ChIP)
H3K27me3, #9733, clone#C36B11, lot# 16, Cell Signaling Technology (1ug per 250k cells in Cut&Run)
H3K9me3, #ab176916, lot# 1011476-14, Abcam (1ug per 250k cells in Cut&Run)
H3K36me3, #61021, clone# 0333, lot# 11721013, Active Motif (1ug per 250k cells in Cut&Run)
SET1A, #ab70378, lot# GR3352449-5, Abcam (1ug per 250k cells in Cut&Run)
SET1A, #50805, clone# E3E2S, lot# 1, Cell Signaling Technology (1:1000 in WB)
SET1B, #44922, clone# D1U5D, lot# 1, Cell Signaling Technology (1:1000 in WB)
CXXC1, #ab198977, lot# GR3245956-8, Abcam (1ug per 250k cells in Cut&Run, 1:1000 in WB)
KMT2A, #A300-086A, lot# 7, Bethyl Laboratories (1ug per 250k cells in Cut&Run)
KMT2A, #14689, clone# D2M7U, lot# 1, Cell Signaling Technology (1:1000 in WB)
KMT2B, #47097, clone# E3M1U, lot# 1, Cell Signaling Technology (1:1000 in WB)
Menin, #A300-105A, lot# 12, Bethyl laboratories (1ug per 250k cells in Cut&Run, 1:1000 in WB)
KMT2D, a kind gift from Dr. Kai Ge's lab (2ug per 10million cells in ChIP, 1:1000 in WB)
GAPDH, #2118, clone# 14C10, lot# 16, Cell Signaling Technology (1:5000 in WB)
Vinculin, #13901, clone# E1E91, lot# 7, Cell Signaling Technology (1:5000 in WB)
PIK3CA, #4249, clone# C73F8, lot# 9, Cell Signaling Technology (1:2000 in WB)
p-AKT S473, #4060, clone# D9E, lot# 24, Cell Signaling Technology (1:2000 in WB)
p-AKT T308, #13038, clone# D25E6, lot#7, Cell Signaling Technology (1:2000 in WB)
AKT, #4691, clone# C67E7, lot#28, Cell Signaling Technology (1:5000 in WB)
p-ERK T202/Y204, #4370, clone# D13.14.4E, lot# 28, Cell Signaling Technology (1:2000 in WB)
RAS, #8832, lot# 9, Cell Signaling Technology (1:500 in WB)
p53, #2524, clone# 1C12, lot# 17, Cell Signaling Technology (1:1000 in WB)
Goat anti-rabbit Alexa Fluor 488, #A11008, lot# 2382186, Thermo Fisher Scientific (1:500 in IF)
Goat anti-rabbit Alexa Fluor 555, #A21428, lot# 2395213, Thermo Fisher Scientific (1:500 in IF)
Goat anti-rabbit Alexa Fluor 633, #A21071, lot# 1073053, Thermo Fisher Scientific (1:500 in IF and Flow)
Goat anti-chicken Alexa Fluor 488, #A11039, lot# 2566343, Thermo Fisher Scientific (1:500 in IF)

Goat anti-mouse Alexa Fluor 488, #A11001, lot# 2379467, Thermo Fisher Scientific (1:500 in IF)
Goat anti-mouse Alexa Fluor 555, #A21422, lot# 2377305, Thermo Fisher Scientific (1:500 in IF and Flow)

Validation

All antibodies except anti-KMT2D antibody were obtained and validated from commercially available sources. All antibodies were applied to mouse cells in this study.
KMT2D antibody was generated by Dr. Kai Ge's lab and validated in KMT2D KO mouse cells (PMID: 37012455).
KRT5: https://www.biolegend.com/en-gb/products/keratin-5-polyclonal-antibody-purified-10956
KRT5: https://www.biolegend.com/fr-ch/products/keratin-5-polyclonal-chicken-antibody-purified-10957
KRT7: https://www.abcam.com/en-us/products/primary-antibodies/cytokeratin-7-antibody-epr17078-cytoskeleton-marker-ab181598
KRT8: https://www.biolegend.com/fr-ch/products/purified-anti-cytokeratin-8-antibody-13078?GroupID=GROUP26
KRT14: https://www.biolegend.com/fr-ch/products/purified-anti-keratin-14-antibody-13379
KRT20: https://www.agilent.com/en/product/immunohistochemistry/antibodies-controls/primary-antibodies/cytokeratin-20-(dako-omnis)-76273
UPK2: https://www.abcam.com/en-us/products/primary-antibodies/uroplakin-ii-upii-antibody-epr18799-ab213655
PTEN: https://www.cellsignal.com/products/primary-antibodies/pten-d4-3-xp-rabbit-mab/9188
GFP: https://www.cellsignal.com/products/primary-antibodies/gfp-d5-1-rabbit-mab/2956
Ki-67: https://www.abcam.com/en-us/products/primary-antibodies/ki67-antibody-sp6-ab16667
SMA: https://www.abcam.com/en-us/products/primary-antibodies/alpha-smooth-muscle-actin-antibody-ab5694
p-EGFR Y845: https://www.cellsignal.com/products/primary-antibodies/phospho-egf-receptor-tyr845-antibody/2231
H-2Kb/Db-APC: https://www.biolegend.com/en-ie/products/apc-anti-mouse-h-2kb-h-2db-antibody-16327
PD-L1-APC: https://www.biolegend.com/fr-ch/products/apc-anti-mouse-cd274-b7-h1-pd-l1-antibody-6655
EpCAM-APC: https://www.thermofisher.com/antibody/product/CD326-EpCAM-Antibody-clone-G8-8-Monoclonal/17-5791-82
H3K4me1: https://www.thermofisher.com/antibody/product/H3K4me1-Antibody-Recombinant-Polyclonal/710795
H3K4me1: https://www.cellsignal.com/products/primary-antibodies/mono-methyl-histone-h3-lys4-d1a9-xp-rabbit-mab/5326
H3K4me2: https://www.thermofisher.com/antibody/product/H3K4me2-Antibody-Recombinant-Polyclonal/710796
H3K4me3: https://www.thermofisher.com/antibody/product/H3K4me3-Antibody-Polyclonal/PA5-27029
H3K27ac: https://www.abcam.com/en-us/products/primary-antibodies/histone-h3-acetyl-k27-antibody-chip-grade-ab4729
H3K27me3: https://www.cellsignal.com/products/primary-antibodies/tri-methyl-histone-h3-lys27-c36b11-rabbit-mab/9733
H3K9me3: https://www.abcam.com/en-us/products/primary-antibodies/histone-h3-tri-methyl-k9-antibody-epr16601-chip-grade-ab176916
H3K36me3: https://www.activemotif.com/catalog/details/61021
SET1A: https://www.abcam.com/en-nc/products/primary-antibodies/hset1-set1-antibody-ab70378
SET1A: https://www.cellsignal.com/products/primary-antibodies/set1a-e3e2s-rabbit-mab/50805
SET1B: https://www.cellsignal.com/products/primary-antibodies/set1b-d1u5d-rabbit-mab/44922
CXXC1: https://www.abcam.com/en-us/products/primary-antibodies/cgbp-antibody-epr19199-chip-grade-ab198977
KMT2A: https://www.thermofisher.com/antibody/product/MLL1-Antibody-Polyclonal/A300-086A
KMT2A: https://www.cellsignal.com/products/primary-antibodies/mll1-d2m7u-rabbit-mab-amino-terminal-antigen/14689
KMT2B: https://www.cellsignal.com/products/primary-antibodies/mll2-kmt2b-e3m1v-rabbit-mab-amino-terminal-antigen/47097
Menin: https://www.thermofisher.com/antibody/product/Menin-Antibody-Polyclonal/A300-105A
GAPDH: https://www.cellsignal.com/products/primary-antibodies/gapdh-14c10-rabbit-mab/2118
Vinculin: https://www.cellsignal.com/products/primary-antibodies/vinculin-e1e9v-xp-rabbit-mab/13901
PIK3CA: https://www.cellsignal.com/products/primary-antibodies/pi3-kinase-p110a-c73f8-rabbit-mab/4249
p-AKT S473: https://www.cellsignal.com/products/primary-antibodies/phospho-akt-ser473-d9e-xp-rabbit-mab/4060
p-AKT T308: https://www.cellsignal.com/products/primary-antibodies/phospho-akt-thr308-d25e6-xp-rabbit-mab/13038
AKT: https://www.cellsignal.com/products/primary-antibodies/akt-pan-c67e7-rabbit-mab/4691
p-ERK T202/Y204: https://www.cellsignal.com/products/primary-antibodies/phospho-p44-42-mapk-erk1-2-thr202-tyr204-d13-14-4e-xp-rabbit-mab/4370
RAS: https://www.cellsignal.com/products/cellular-assay-kits/active-ras-detection-kit/8821
p53: https://www.cellsignal.com/products/primary-antibodies/p53-1c12-mouse-mab/2524
Goat anti-rabbit Alexa Fluor 488: https://www.thermofisher.com/antibody/product/Goat-anti-Rabbit-IgG-H-L-Cross-Adsorbed-Secondary-Antibody-Polyclonal/A-11008
Goat anti-rabbit Alexa Fluor 555: https://www.thermofisher.com/antibody/product/Goat-anti-Rabbit-IgG-H-L-Cross-Adsorbed-Secondary-Antibody-Polyclonal/A-21428
Goat anti-rabbit Alexa Fluor 633: https://www.thermofisher.com/antibody/product/Goat-anti-Rabbit-IgG-H-L-Highly-Cross-Adsorbed-Secondary-Antibody-Polyclonal/A-21071
Goat anti-chicken Alexa Fluor 488: https://www.thermofisher.com/antibody/product/Goat-anti-Chicken-IgY-H-L-Secondary-Antibody-Polyclonal/A-11039
Goat anti-mouse Alexa Fluor 488: https://www.thermofisher.com/antibody/product/Goat-anti-Mouse-IgG-H-L-Cross-Adsorbed-Secondary-Antibody-Polyclonal/A-11001
Goat anti-mouse Alexa Fluor 555: https://www.thermofisher.com/antibody/product/Goat-anti-Mouse-IgG-H-L-Cross-Adsorbed-Secondary-Antibody-Polyclonal/A-21422

Animals and other research organisms

Policy information about studies involving animals; ARRIVE guidelines recommended for reporting animal research, and Sex and Gender in Research

Laboratory animals

1. Tmprss2-CreERT2-IRES-nlsEGFP (Tmprss2tm1.1(cre/ERT2)Ychen, MGI:5911389) was generated in our lab.
2. Pten flox (Ptentm2.1Ppp, MGI:2679886).
3. Kmt2c flox strain with Exon 3 flanked by LoxP sites was obtained from the Sarat Chandarlapaty's lab.
4. Kmt2d flox strain with Exon 50-51 flanked by LoxP sites was obtained from the Kai Ge's lab.
5. Rosa26-CAG-LSL-EYFP strain was obtained from Jackson Laboratory (B6.Cg-Gt(ROSA)26Sortm3(CAG-EYFP)Hze, Stock No: 007903).
6. Female NOD-SCID mice (6-8 weeks old) were obtained from Jackson Laboratory (Strain/Stock: NOD.CB17-Prkdc<scid>, Stock #:

001303).
To induce gene knockout in GEMM, tamoxifen was given to mice of 6-12 weeks old. Intravesical delivery of 4OHT and adenovirus were performed on mice of 6-12 weeks old.
Mice were maintained under 12h light/dark cycle (lights on/off at 6am/pm), with controlled temperature and humidity, and with access to regular chow and sterilized water ad libitum.

Wild animals: Wild animals were not used in the study.

Reporting on sex: This study included both male and female mice as detailed in the figures or figure legends.

Field-collected samples: No field collected samples were used in the study.

Ethics oversight: Mouse experiments were conducted under protocol 11-12-027 approved by Institutional Animal Care and Use Committee (IACUC) of MSKCC, New York.

Note that full information on the approval of the study protocol must also be provided in the manuscript.

Plants

Seed stocks: *Report on the source of all seed stocks or other plant material used. If applicable, state the seed stock centre and catalogue number. If plant specimens were collected from the field, describe the collection location, date and sampling procedures.*

Novel plant genotypes: *Describe the methods by which all novel plant genotypes were produced. This includes those generated by transgenic approaches, gene editing, chemical/radiation-based mutagenesis and hybridization. For transgenic lines, describe the transformation method, the number of independent lines analyzed and the generation upon which experiments were performed. For gene-edited lines, describe the editor used, the endogenous sequence targeted for editing, the targeting guide RNA sequence (if applicable) and how the editor was applied.*

Authentication: *Describe any authentication procedures for each seed stock used or novel genotype generated. Describe any experiments used to assess the effect of a mutation and, where applicable, how potential secondary effects (e.g. second site T-DNA insertions, mosiacism, off-target gene editing) were examined.*

ChIP-seq

Data deposition

☒ Confirm that both raw and final processed data have been deposited in a public database such as GEO.

☒ Confirm that you have deposited or provided access to graph files (e.g. BED files) for the called peaks.

Data access links
May remain private before publication.
https://www.ncbi.nlm.nih.gov/geo/query/acc.cgi (GSE180947, GSE236370, GSE264514)

Files in database submission
GSM8219288 RNAseq_WT_DMSO_Rep1
GSM8219289 RNAseq_WT_DMSO_Rep2
GSM8219290 RNAseq_WT_DMSO_Rep3
GSM8219291 RNAseq_WT_MI503_Rep1
GSM8219292 RNAseq_WT_MI503_Rep2
GSM8219293 RNAseq_WT_MI503_Rep3
GSM8219294 RNAseq_dKO_DMSO_Rep1
GSM8219295 RNAseq_dKO_DMSO_Rep2
GSM8219296 RNAseq_dKO_DMSO_Rep3
GSM8219297 RNAseq_dKO_MI503_Rep1
GSM8219298 RNAseq_dKO_MI503_Rep2
GSM8219299 RNAseq_dKO_MI503_Rep3
GSM8219300 ATACseq_WT_Rep1
GSM8219301 ATACseq_WT_Rep2
GSM8219304 ATACseq_dKO_Rep1
GSM8219305 ATACseq_dKO_Rep2
GSM8219308 ChIPseq_H3K27ac_WT_Rep1
GSM8219309 ChIPseq_H3K27ac_WT_Rep2
GSM8219311 ChIPseq_H3K27ac_dKO_Rep1
GSM8219312 ChIPseq_H3K27ac_dKO_Rep2
GSM8219315 CutRun_Kmt2a_WT_Rep1
GSM8219316 CutRun_Kmt2a_WT_Rep2
GSM8219318 CutRun_Kmt2a_dKO_Rep1
GSM8219319 CutRun_Kmt2a_dKO_Rep2
GSM8219320 CutRun_H3K4me1_WT_Rep1
GSM8219321 CutRun_H3K4me1_WT_Rep2
GSM8219330 CutRun_H3K4me1_dKO_Rep1
GSM8219331 CutRun_H3K4me1_dKO_Rep2
GSM8219323 CutRun_H3K4me2_WT_1
GSM8219324 CutRun_H3K4me2_WT_2
GSM8219332 CutRun_H3K4me2_dKO_1

GSM8219333 CutRun_H3K4me2_dKO_2
GSM8219327 CutRun_H3K27me3_WT_Rep1
GSM8219328 CutRun_H3K27me3_WT_Rep2
GSM5478809 CutRun_H3K27me3_WT_Rep3
GSM8219335 CutRun_H3K27me3_dKO_Rep1
GSM8219336 CutRun_H3K27me3_dKO_Rep2
GSM5478810 CutRun_H3K27me3_dKO_Rep3
GSM5478807 WT_CutRun_H3K4me3_Rep1
GSM7528793 WT_CutRun_H3K4me3_Rep2
GSM5478808 dKO_CutRun_H3K4me3_Rep1
GSM7528794 dKO_CutRun_H3K4me3_Rep2
GSM7528795 WT_MI503_CutRun_H3K4me3
GSM7528796 dKO_MI503_CutRun_H3K4me3
GSM5478811 WT_CutRun_Menin_Rep1
GSM7528797 WT_CutRun_Menin_Rep2
GSM5478812 dKO_CutRun_Menin_Rep1
GSM7528798 dKO_CutRun_Menin_Rep2
GSM7528799 WT_MI503_CutRun_Menin
GSM7528800 dKO_MI503_CutRun_Menin
GSM5478815 WT_scRNAseq_Rep1
GSM5478816 WT_scRNAseq_Rep2
GSM5478817 WT_scRNAseq_Rep3
GSM5478818 WT_scRNAseq_Rep4
GSM5478819 dKO_scRNAseq_Rep1
GSM5478820 dKO_scRNAseq_Rep2
GSM5478821 dKO_scRNAseq_Rep3
GSM7528773 WT_PROcap_Rep1
GSM7528774 WT_PROcap_Rep2
GSM7528775 dKO_PROcap_Rep1
GSM7528776 dKO_PROcap_Rep2
GSM7528777 WT_Kmt2d_ChIP_Rep1
GSM7528778 WT_Kmt2d_ChIP_Rep2
GSM7528779 dKO_Kmt2d_ChIP_Rep1
GSM7528780 dKO_Kmt2d_ChIP_Rep2
GSM7528781 WT_ChIP_Input_Rep1
GSM7528782 WT_ChIP_Input_Rep2
GSM7528783 dKO_ChIP_Input_Rep1
GSM7528784 dKO_ChIP_Input_Rep2
GSM7528801 WT_CutRun_Cxxc1_Rep1
GSM7528802 WT_CutRun_Cxxc1_Rep2
GSM7528803 dKO_CutRun_Cxxc1_Rep1
GSM7528804 dKO_CutRun_Cxxc1_Rep2
GSM7528805 WT_CutRun_Set1a_Rep1
GSM7528806 WT_CutRun_Set1a_Rep2
GSM7528807 dKO_CutRun_Set1a_Rep1
GSM7528808 dKO_CutRun_Set1a_Rep2
GSM7528809 WT_CutRun_H3K9me3
GSM7528810 dKO_CutRun_H3K9me3
GSM7528811 WT_CUTnRUN_H3K36me3
GSM7528812 dKO_CUTnRUN_H3K36me3

Genome browser session
(e.g. UCSC)

Not applicable.

Methodology

Replicates

WT and dKO, scRNA-seq (n=4 mice in WT, n=3 mice in Kmt2c/d dKO)
WT and dKO, Bulk RNA-seq, DMSO (3 replicates)
WT and dKO, Bulk RNA-seq, MI503 (3 replicates)
WT and dKO, PRO-cap (2 replicates)
WT and dKO, KMT2D (2 replicates in ChIP-seq)
WT and dKO, H3K4me1 (2 replicates in Cut&Run)
WT and dKO, H3K4me2 (2 replicates in Cut&Run)
WT and dKO, H3K4me3 (2 replicates in Cut&Run)
WT and dKO, H3K27ac (2 replicate in ChIP-seq)
WT and dKO, ATAC-seq (3 replicates)
WT and dKO, H3K27me3 (3 replicate in Cut&Run)
WT and dKO, H3K9me3 (1 replicate in Cut&Run)
WT and dKO, H3K36me3 (1 replicate in Cut&Run)
WT and dKO, SET1A (2 replicates in Cut&Run)
WT and dKO, CXXC1 (2 replicates in Cut&Run)
WT and dKO, KMT2A (2 replicates in Cut&Run)
WT and dKO, Menin (2 replicate in Cut&Run)
WT and dKO, Menin_MI503 (1 replicate in Cut&Run)
WT and dKO, H3K4me3_MI503 (1 replicate in Cut&Run)

	WT and dKO, Input (3 replicates in ChIP-seq)
Sequencing depth	In poly-A bulk RNA-seq, paired end 50bp or 100bp, 30-40 million reads.<br>In scRNA-seq, paired end 28/91, median reads count 12,984 per cell.<br>In PRO-cap, paired end 150bp, 60-70 million reads.<br>In ATAC-seq, paired end 50bp, 40-50 million reads.<br>In ChIP-seq, paired end 50bp or 100bp, 30-40 million reads, or more.<br>In Cut&Run, paired end 50bp or 100bp, 10-20 million reads, or more.
Antibodies	H3K4me1, #710795, Thermo Fisher Scientific (1ug per 250k cells in Cut&Run)<br>H3K4me2, #710796, Thermo Fisher Scientific (1ug per 250k cells in Cut&Run)<br>H3K4me3, #PA57-27029, Thermo Fisher Scientific (1ug per 250k cells in Cut&Run)<br>H3K27ac, #ab4729, Abcam (2ug per 10million cells in ChIP)<br>H3K27me3, #9733, Cell Signaling Technology (1ug per 250k cells in Cut&Run)<br>H3K9me3, #ab176916, Abcam (1ug per 250k cells in Cut&Run)<br>H3K36me3, #61021, Active Motif (1ug per 250k cells in Cut&Run)<br>SET1A, #ab70378, Abcam (1ug per 250k cells in Cut&Run)<br>CXXC1, #ab198977, Abcam (1ug per 250k cells in Cut&Run)<br>KMT2A, #A300-086A, Bethyl Laboratories (1ug per 250k cells in Cut&Run)<br>Memin, #A300-115A, Bethyl laboratories (1ug per 250k cells in Cut&Run)<br>KMT2D, a kind gift from Dr. Kai Ge's lab (2ug per 10million cells in ChIP)
Peak calling parameters	MACS3 (3.0.0) was used for peak calling in ChIP-seq, ATAC-seq, Cut&Run, -q e-2.<br>PINTS (1.1.8) was used for peak calling in PRO-cap, default parameters.
Data quality	All peaks in our study were called with FDR < 0.01.
Software	scRNA-seq data were processed with Cell Ranger (7.0.0). True cells were identified using scCB2 package (1.14.0). Putative doublets were detected and filtered out using doublet detection package (4.2). Downstream analyses and figure plotting were performed using Scanpy (1.6.1). Differentially expressed genes were compared with MAST package (1.30.0). Imputated data were used to generate heatmap using MAGIC (3.0.0) package.<br>RNA-seq data were processed with STAR (2.7.10b);<br>PRO-cap data were mapped with STAR (2.7.10b). Peaks were called with PINTS 1.1.8 (pints_caller). Bigwig files of plus and minus strand alignments were generated with PINTS 1.1.8 (pints_visualizer);<br>Data from ATAC-seq, ChIP-seq, Cut&Run were trimmed with trim_galore and mapped to GRCm38(mm10) using bowtie2 (2.4.5). Duplicates were marked with samblaster (0.1.26). Mapping quality was analyzed with qualimap (2.2.2-dev). Peaks were called using MACS3 (3.0.0). Bigwig files were generated with bamCoverage (3.5.1); Read counts were measured with featureCounts (v2.0.1); Heatmap and aggregation plots were generated using deepTools (3.5.1).<br>In KMT2D ChIP-seq, Sicer2 (1.0.3) (sicer_df) was used to call differential peaks between WT and dKO conditions;<br>Homer (4.11.1) was employed to merge (Homer mergePeaks) and annotate (Homer annotatepeaks.pl) peaks from PRO-cap, ATAC-seq, ChIP-seq, and Cut&Run;<br>ChromHMM (v1.25) LearnModel was performed to investigate chromatin state in WT and dKO cells. ChromHMM OverlapEnrichment was conducted to compare enrichment of chromatin states at given coordinates.

Flow Cytometry

Plots

Confirm that:

☒ The axis labels state the marker and fluorochrome used (e.g. CD4-FITC).

☒ The axis scales are clearly visible. Include numbers along axes only for bottom left plot of group (a 'group' is an analysis of identical markers).

☒ All plots are contour plots with outliers or pseudocolor plots.

☒ A numerical value for number of cells or percentage (with statistics) is provided.

Methodology

Sample preparation	For urothelial cell sorting, bladders were dissected out and minced with scalpel, and then processed for 1h digestion with collagenase/hyaluronidase (#07912, STEMCELL Technologies) and 15min digestion with TrypLE (#12605010, Gibco). Dissociated were staining with EpCAM-APC (#17579180, Thermo Fisher Scientific) for 30min on ice. Live single urothelial cells were sorted out by flow cytometry as DAPI-/EpCAM+/nlsEGFP+.<br>For intracellular flow cytometry analysis, organoids were digested with Dispase for 30min and then further dissociated into single cells with TrypLE for 15min on a shaker in the cell culture incubator. Single urothelial cells were then fixed with 4% PFA for 10min and permeabilized with 0.5% Triton-X 100 for 10min. Primary antibodies against KRT5 and KRT8, secondary antibodies Alexa fluor 633 conjugated goat anti-rabbit and Alexa fluor 555 conjugated goat anti-mouse were then applied in order, 30min on ice.<br>For cell surface flow cytometry analysis, fluorescence-conjugated antibodies against H-2Kb/H-2Db-APC (#114614, Biolegend) and PD-L1-APC (#124311, Biolegend) were directly stained with viable cells, 30min on ice.
Instrument	BD LSRFortessa, BD FACSymphony S6 Cell Sorter

Software	Data were collected with BD FACSDiva (v6.2) software and analyzed with FlowJo (10.7.1).
Cell population abundance	In the cell sorting, viable urothelial cells were determined by double positivity of pan-epithelial cell marker EpCAM-APC and nlsEGFP expression.
Gating strategy	FSC and SSC were used to gate dissociated single cells. Cell viability dye (DAPI) was used to identify viable single cells. Isotype control antibodies were used to define background. For cell sorting, viable urothelial cells were gated as EpCAM-APC and nlsEGFP double positive. For cell surface or intracellular marker analyses, the mean fluorescence intensity (MFI) of indicated markers were analyzed on urothelial cells.

☒ Tick this box to confirm that a figure exemplifying the gating strategy is provided in the Supplementary Information.
