# Supplementary figures and images for: Loss of Kmt2c or Kmt2d primes urothelium for tumorigenesis and redistributes KMT2A–menin to bivalent promoters
Source: Nat Genet. 2025 Jan 13;57(1):165–79. doi: 10.1038/s41588-024-02015-y (PMC11735410; doi:10.1038/s41588-024-02015-y)

Extended Data Fig. 4b

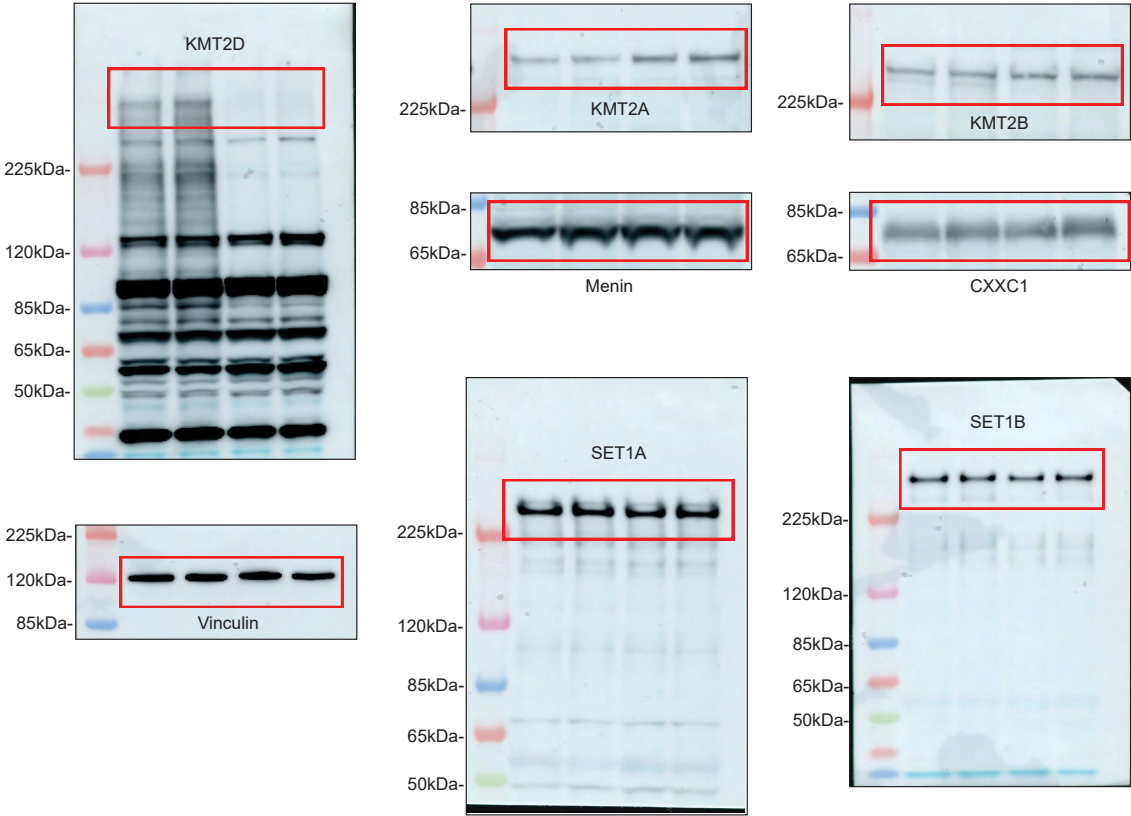

Extended Data Fig. 4c

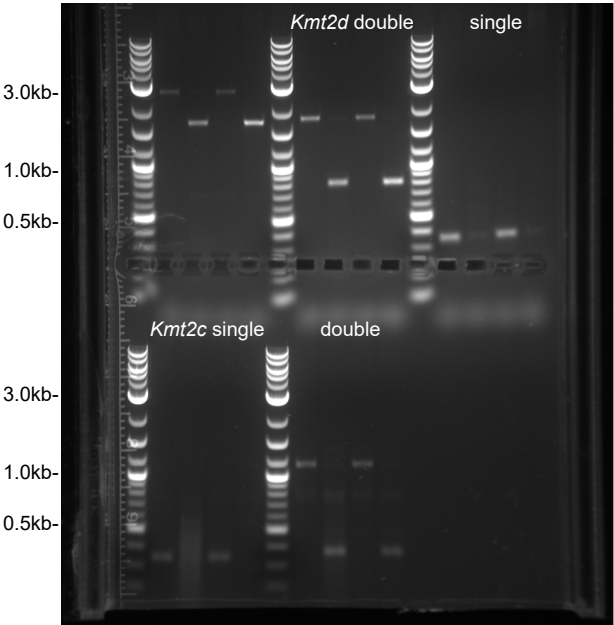

Supplement: Supplementary file 14 — Uncropped gel and membrane. [file 41588_2024_2015_MOESM14_ESM.pdf]
