# Supplementary material for: Loss of Kmt2c or Kmt2d primes urothelium for tumorigenesis and redistributes KMT2A–menin to bivalent promoters
Source: Nat Genet. 2025 Jan 13;57(1):165–79. doi: 10.1038/s41588-024-02015-y (PMC11735410; doi:10.1038/s41588-024-02015-y)

Extended Data Fig. 10a

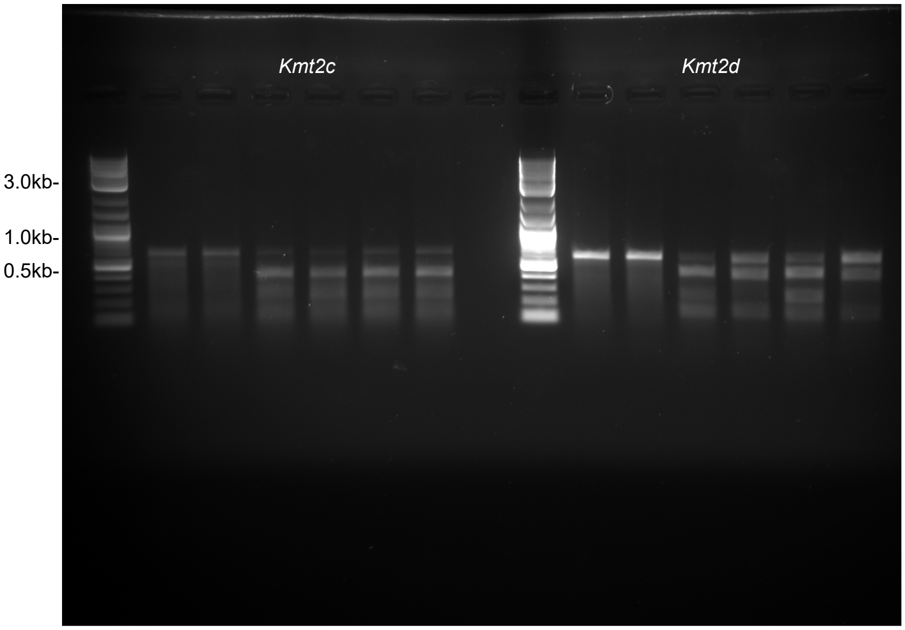

Extended Data Fig. 10b

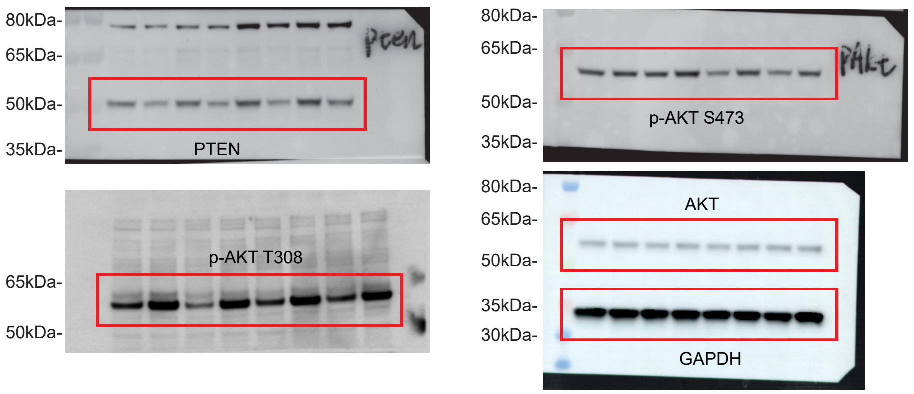

Extended Data Fig. 10c

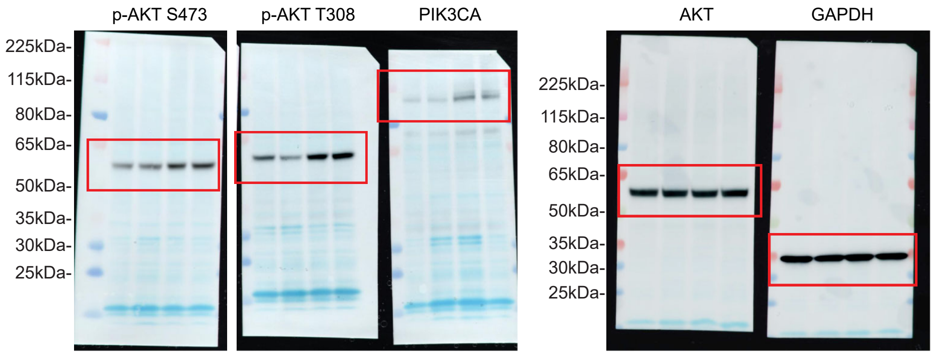

Extended Data Fig. 10d

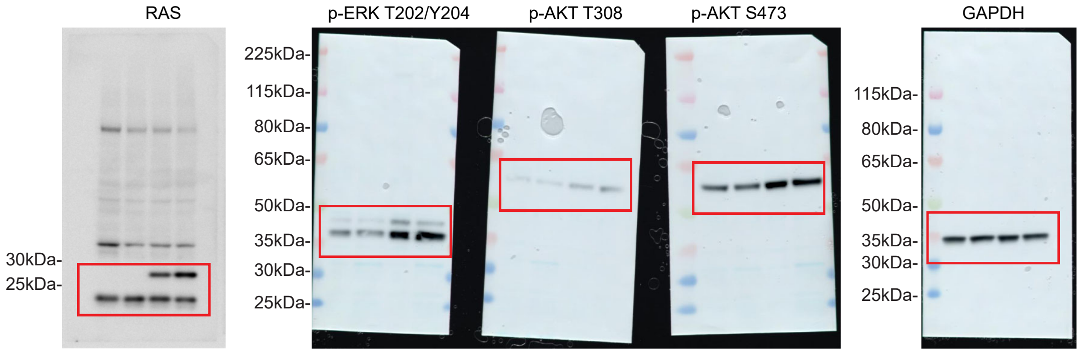

Extended Fig. 10e

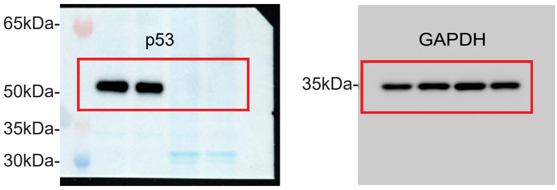

Extended Data Fig. 10f

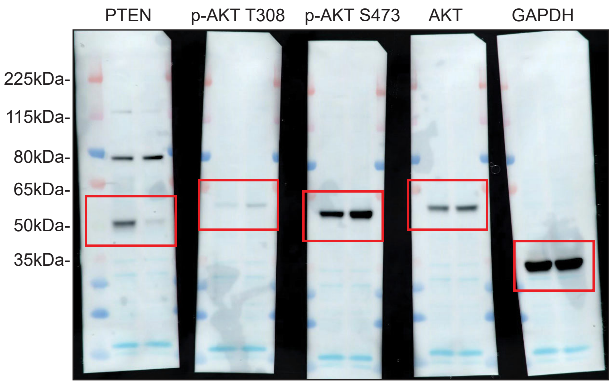

Supplement: Supplementary file 18 — Uncropped gel and membrane. [file 41588_2024_2015_MOESM18_ESM.pdf]
